# Quantifying the influence of vocational education and training with text embedding and similarity-based networks

**DOI:** 10.1371/journal.pone.0329405

**Published:** 2025-08-21

**Authors:** Hyeongjae Lee, Inho Hong

**Affiliations:** 1 Graduate School of Data Science, Chonnam National University, Gwangju, Korea; 2 Center for Humans and Machines, Max Planck Institute for Human Development, Berlin, Germany; University of Milano–Bicocca: Universita degli Studi di Milano-Bicocca, ITALY

## Abstract

Assessing the potential influence of Vocational Education and Training (VET) courses on creating job opportunities and nurturing work skills has been considered challenging due to the ambiguity in defining their complex relationships and connections with the local economy. Here, we quantify the potential influence of VET courses and explain it with future economy and specialization by constructing a network of more than 17,000 courses, jobs, and skills in Singapore’s SkillsFuture data based on their text similarities captured by a text embedding technique, Sentence Transformer. We find that VET courses associated with Singapore’s 4th Industrial Revolution economy demonstrate higher influence than those related to other future economies. The course influence varies greatly across different sectors, attributed to the level of specificity of the skills covered. Lastly, we show a notable concentration of VET supply in certain occupation sectors requiring general skills, underscoring a disproportionate distribution of education supply for the labor market.

## Introduction

Vocational Education and Training (VET) enhances human capital development by directly connecting with technological standards, the organization of the production process, and culturally specific work divisions, all of which necessitate work-related upper secondary and post-secondary qualifications [1]. From the individual perspective, VET not only facilitates smooth entry into the labor market early in an employee’s career but also supports long-term career stability by enhancing adaptability to rapid technological and structural changes in the economy through reskilling and upskilling [2–4].

Despite its importance in the labor market, quantifying the influence of VET and assessing its effectiveness remains challenging due to its complex relationships with local environments. Unlike a standardized or global model, VET often functions as a subsystem shaped by stimuli at the country or regional level [5–7]. An intriguing indicator proposed to measure VET effectiveness is vocational or occupational specificity, focusing on evaluating VET in the context of School-to-Work linkage [8, 9]. While this index provides valuable insights, a practical challenge arises in profiling and tracking the education-to-employment pathways of individuals who have completed specific VET programs [10, 11]. This challenge is particularly pronounced given the contemporary labor market trend where individuals’ school-to-work transitions and career pathways throughout their lifespan are increasingly diversified [12, 13].

As numerous countries are making substantial investments in VET to ensure that their labor forces are well-equipped with the necessary skills and readiness for emerging industries [14, 15], another crucial aspect of VET programs worth investigating is an imbalance between supply and demand in the VET market [16]. A disparity between the supply and demand of VET provisions turns out to be a key factor in hindering educational equality, which is closely related to the development of a regional economy [17]. In that sense, detecting any imbalance in the VET market of a region holds implications for informed policy-making in addressing educational challenges, leading to the sustainable development of the region [18, 19].

Analyzing occupations, human capital, and VET courses as an interconnected network enables the examination of their relationships and the complementarity among various VET programs [20]. Network analysis has been implemented for exploring the interconnections of human capital entities over different labor markets [21]. A network analysis of job roles and their associated skills across industries in Singapore revealed distinct clusters of similar positions and interrelated skills that provide intuitive visualizations to inform job market trends and guide both job seekers and employers [22]. Utilizing the co-occurrence of skills among job seekers and providers from O*NET data, researchers explained the economic inequality and the hollowing of the middle class with the polarization between cognitive and physical skills in high-wage workers and low-wage workers within the constructed networks [23]. Similarly, a collaboration network of workers with different educational backgrounds revealed that co-workers who are more synergistic are more likely to substitute for one another [24]. As such, while the labor market and human capital domain have been actively explored, the VET domain largely remains unexplored. This gap arises from the practical issue of finding a suitable dataset that captures the direct connections between VET courses, occupations, and skills as individual entities.

Recent advancements in Natural Language Processing (NLP) can provide an alternative approach to overcome this difficulty [25, 26]. To be specific, contextual embedding using unstructured textual data from various sources has enabled researchers to access the semantic dimension of socio-economic entities and explore their relationships within high-dimensional vector space [27, 28]. For instance, trained on 850 billion words in English-language books from the Google n-grams dataset, historical shifts in different social-cultural groups are traced by comparing the top words associated with each group from the embedded space [29]. Likewise, utilizing a Google news dataset and a pre-trained w2vNEWs model, gender bias has been revealed through vector analogy between English vocabularies [30]. Generating a culture dictionary and scoring words based on word representation trained from earning call transcripts, a study has attempted to measure corporate culture and demonstrate its association with business outcomes [31].

When it comes to exploring the semantic relations between entities in human capital and labor market research, the heterogeneous entities of industries, occupations, skills, and firms can be mapped onto a unified labor space using the recent BERT model [32, 33]. Additionally, by focusing on university course syllabi and measuring their distance from frontier knowledge within the embedded space, the innovation gap across higher educational institutions has been explained with the academic and economic outcomes of graduates [34]. The recently publicized dataset relating higher education curricula and skills based on text embedding suggests a new possibility to quantify the relationship between VET courses and skills [35]. These cases highlight the potential of NLP in extracting meaningful insights from unstructured textual data across diverse fields, showcasing its versatility and applicability in understanding complex dynamics in the education-labor market.

Drawing on textual data from descriptions of VET courses, skills, and occupations provided by Singapore’s SkillsFuture initiative, this study employs the Sentence Transformer model [36] to represent each unit of analysis as a single vector. A network is subsequently constructed on the basis of semantic similarities among these vectors [37, 38]. Although not identically the same, there is a similar technique called Semantic Network Analysis (SNA) [39]. SNA is a technique to discover semantic structures in texts by constructing a co-occurrence matrix and a network based on the words in texts. Semantic network analysis has been applied to understand how user opinions and experiences are communicated within online communities or how different concepts are represented or related to each other [40, 41]. While similar, this study differs in that, instead of constructing a co-occurrence matrix based on word occurrences, it builds the network by directly measuring the semantic similarity among VET course, skill, and occupation units.

From this background, the study is designed to address the following research questions. First, the study examines whether a network of VET courses can be constructed based on text similarity by applying NLP techniques to identify semantic relationships among courses using their descriptions and related documents. Second, the study aims to develop an index to quantify the influence of VET courses and compare it across sectors, identifying key contributing factors. Last, the study investigates whether an imbalance exists in the distribution of VET programs across Singapore’s industries by assessing if these programs align with industry demand and examining the implications of any disparities for education and economic development.

To address these questions, our analysis of Singapore’s SkillsFuture data quantitatively evaluates the influence of VET entities through a novel course influence index, derived from a network of VET courses based on text similarity. This network is built by embedding course, skill, and job descriptions using a pre-trained Sentence Transformer, constructing a bipartite network of VET courses and skills, which is then projected into a monopartite VET course network, where connections indicate significant similarity. The course influence index is calculated as the coverage ratio (the number of distinct skills) to degree centrality (the number of similar courses). This metric considers both the breadth of skills a course offers and the redundancy of VET courses, ensuring a balanced evaluation of educational coverage and overlap. In addition, we analyze the relationship between course influence, skill diversity, and occupational transferability by incorporating these factors into a regression model.

To examine disparities in the distribution of VET courses, we construct a tripartite network linking courses, skills, and occupations based on textual similarity. This network is then transformed into a bipartite course-occupation network, allowing an assessment of the distribution of VET course offerings across job categories. Finally, leveraging this network, we introduce an occupational course supply index (i.e.,OCS index) to measure how well the supply of VET courses aligns with industry demand.

## Materials and methods

### SkillsFuture course dataset

The study employs textual data comprising the learning objectives and course descriptions of VET courses (n=13,694), along with associated information on skills (n=2,580) and occupations (n=1,456), all sourced from SkillsFuture Singapore. Launched in 2015 by the Singapore government, SkillsFuture is a national initiative designed to empower Singaporeans through skill development and lifelong learning [42]. It fosters a culture of continuous learning and professional development by offering a comprehensive suite of programs and resources. As a central component of the human capital investment, every Singapore citizen aged 25 and above is eligible for claiming SkillsFuture credits, which can be used to enroll in various skills-related courses available on the official portal at https://www.myskillsfuture.gov.sg/content/portal/en/training-exchange/course-landing.html. The initiative also provides career guidance, advisory services, and a curated selection of training programs tailored to meet the evolving demands of the modern workforce. The SkillsFuture portal features courses spanning various training areas aligned with major national industries and delivers detailed information on curricula, training agencies, and course objectives via Skillsfuture API.

In the study, VET courses are defined as skills-related courses offered under the SkillsFuture initiative and delivered by institutions including the Institute of Technical Education (ITE) and accredited private training agencies. Of the 29,917 courses available as of 19 September 2023, only the 13,694 courses with syllabi in English (with those offered in Chinese excluded to suppress language dependency of embedding vectors) were included as the final sample. This means that 54.22% of the total courses with Chinese-language syllabi were excluded from the analysis to ensure linguistic consistency and reduce potential bias in the embedding process. Among 57 total categories, the top 15 representative fields reveal that “Information and Communications” accounts for the highest share at 16.68%, followed by “Engineering”(9.11%), “Business Management” (6.42%), and “Healthcare” (6.05%) (see S1 Fig and S1 Table for more details). For the analysis, the objective and content of each course were utilized as the textual information.

### SkillsFuture skills frameworks

In addition to the course data, this study leverages skills and occupations datasets from the SkillsFuture Framework, which provides a structured guide for workforce development by defining key skills, job roles, and training pathways. Developed through collaboration between the government, industry, and professional bodies, the framework is continuously refined by SkillsFuture Singapore using labor market intelligence and expert validation.

The original skill dataset includes skill type, skill code, industrial sector, skill category, skill title, and description, along with proficiency levels and knowledge/ability indicators. These skills encompass both core professional competencies and specialized technical expertise across various industries. Since a single skill, identified by its title, can be associated with multiple proficiency-specific codes (e.g., “ACC-AUD-4003-1.1”, “ACC-AUD-5003-1.1” for Audit Frameworks), these were aggregated into a single skill entity. Consequently, only skill title and skill category were used for analysis in this study (see S1 Table for mores details).

When it comes to job entities, their descriptions and key tasks serve as their textual representation, while the sector field, which identifies the relevant industry, is included in the analysis. Additionally, wage information is used to explore the relationship between salaries and the proposed new index.

### Future economy and skill transferability

Transferable skills are abilities that can be applied across various situations and aspects of life [43]. In the context of the SkillsFuture dataset, transferability refers to the number of unique occupations that require skills relevant to four distinct future economy sectors: the green economy [44], digital economy [45], care economy [46], and the Fourth Industrial Revolution economy, as defined by SkillsFuture [47]. For each sector, SkillsFuture Singapore identifies priority skills for emerging industry domains (e.g., “Energy, Resource Circularity and Decarbonisation” or “Product Innovation and Quality Management”) and evaluates their transferability based on the number of unique job roles requiring each skill, as derived from job postings analyzed from 2022 to 2023 (see S1 Table for details).

### Constructing the course network based on text similarity

**Fig 1:**
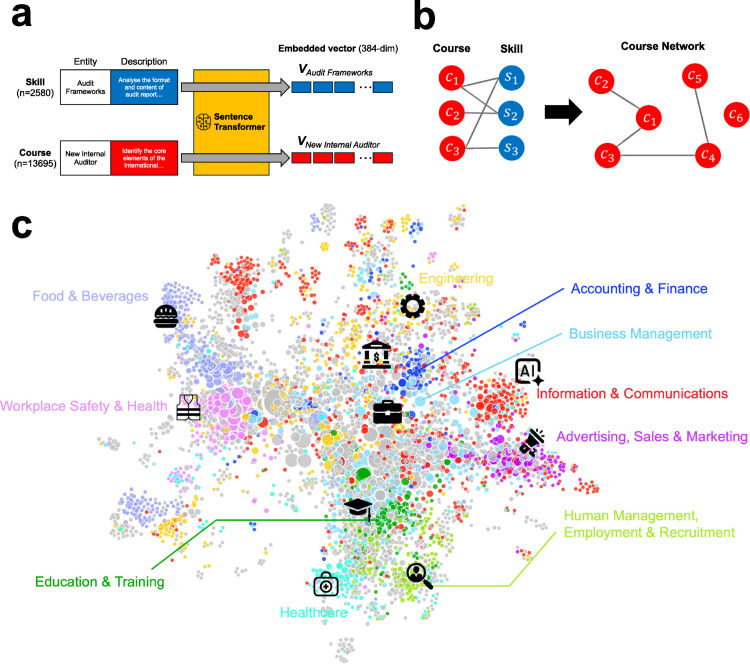
VET course network. **a**. Schematic of text embedding. The diagram shows the embedding process, where textual descriptions of skills and VET courses are transformed into vector representations using a pre-trained Sentence Transformer model. **b**. Schematic of constructing a course network from the course-skill bipartite network. The course network is the co-skill network of VET courses. **c**. Illustration of the course network. Each node represents a VET course that has more than one neighbor from the course-skill bipartite network. The node color signifies the VET course category, while the node size demonstrates the degree centrality. Links are omitted in the visualization for simplicity. The colored nodes represent the top 10 categories with the highest number of VET courses, highlighting the most dominant sectors in SkillsFuture Singapore.

We first built a bipartite network of VET courses and skills by connecting VET courses with skills based on their calculated textual similarities to create a monopartite VET course network ultimately. This involved embedding text descriptions of each entity into 384-dimensional vectors using the pre-trained Sentence Transformer model without fine-tuning [36] as in Fig 1a. Specifically, we utilized an “all-MiniLM-L6-v2” model, which is optimized for downstream tasks such as information retrieval, clustering, and sentence similarity measurement. The model delivers robust semantic representations that capture subtle nuances in short paragraphs, making it suitable for our analysis [48].

We followed the standard guidelines recommended by Hugging Face for tokenization, which involves both padding and truncation. For each course description, we encoded inputs with a maximum of 256 word token size: shorter texts were padded to reach this length, while longer texts were truncated. The linkage between a VET course and a work skill was established by computing their cosine similarity with a threshold of 0.6. For the bipartite network of courses and skills, the adjacency matrix *A*_*cs*_ takes a value of 1 when a course *c* effectively covers skill *s*, and 0 otherwise as

$$A_{cs}=\left\{ \begin{array}{cc} 1 & \text{if }\frac{V_{c}\cdot V_{s}}{\|V_{c}\|\|V_{s}\|}\geq 0.6,\\ 0 & \text{otherwise}, \end{array}\right.$$ (1)

where $V_{c}$ and $V_{s}$ are the embedding vectors of course *c* and skill *s*, respectively. We set this threshold of 0.6 by taking the inflection point in the function of the giant component fraction and threshold (see S2 Fig for the function of the giant component fraction and threshold). Subsequently, the resulting bipartite network was projected to generate a VET course network, where nodes are VET courses and links are connected between two VET courses that are connected to the same skills in the bipartite network (Fig 1b).

### Constructing the course-occupation network

We constructed a course-occupation network that links VET courses with occupations based on shared skills. Initially, we assembled a tripartite network consisting of courses, skills, and occupations by integrating the course-skill bipartite network with a skill-occupation network. In constructing the skill-occupation network, skills and occupations were connected in the same manner as courses and skills: cosine similarity between their respective embedding vectors was computed, and a threshold of 0.6 was applied to establish a link. Finally, we projected the tripartite network onto a course-occupation network by connecting each course to any occupation that could be reached via an intermediate skill.

### VET specificity

The proposed index, VET specificity, serves as a proxy to evaluate the extent to which VET courses within the network specialize in specific skills, as adopted from the studies on economic complexity [49–51]. Leveraging *A*_*cs*_, the number of occupations and skills covered by each course (i.e. diversification), *d*_*c*_, and the number of different courses that cover a particular skill (i.e. ubiquity), *u*_*s*_, can be computed as $d_{c}=\sum_{s}A_{cs}$ and $u_{s}=\sum_{c}A_{cs}$, respectively. Then, ${\bar{u}}_{c}$ represents course *c*’s average ubiquity of the connected skills as

$${\bar{u}}_{c}=\frac{1}{N_{c}}\sum_{s}A_{cs}u_{s},$$ (2)

where *N*_*c*_ is the number of skills connected to course *c*. Using this, we define VET specificity as the inverse of the average ubiquity as $S_{c}=1/{\bar{u}}_{c}$ which measures how much a course is connected to less ubiquitous (i.e., more specific) skills.

### VET transferability for courses and occupations on future economy

We computed the transferability *T*_*c*,*e*_ for each VET course node *c* and each future economy sector *e* by aggregating the transferability values of every skill node neighbor connected to individual course nodes within the constructed VET network as

$$T_{c,e}=\sum_{s}A_{cs}T_{s,e},$$ (3)

where *T*_*s*,*e*_ is the transferability value of skill *s* for future economy sector *e*, provided by the SkillsFuture dataset. A higher transferability value for a specific course node implies that the course is more likely to contribute to generating more employment related to that particular future economy.

Additionally, we analyzed the relationship between course supply and future economies by computing occupational transferability values for each sector. The occupational transferability *T*_*o*,*e*_ for occupation *o* and future economy *e* was obtained by aggregating the skill transferability *T*_*s*,*e*_ over the skills *s* linked to occupation *o* as

$$T_{o,e}=\sum_{s}A_{os}T_{s,e},$$ (4)

where *A*_*os*_ is the adjacency matrix of the bipartite occupation-skill network. For each of the four future economies, sector-level transferability was calculated as the average occupational transferability across all occupations in that sector.

### VET skill diversity

The diversity of each VET course is expressed through the Shannon entropy value for skill categories connected to each course node. The entropy value of a course gets higher when the categories of skills connected to the course are more evenly distributed. Here the skill diversity *H*_*c*_ of course *c* is defined as

$$H_{c}=-\sum_{x}p_{c}(x)\log p_{c}(x),$$ (5)

where *p*_*c*_(*x*) represents the ratio of the number of skills in skill category *x* to the total number of skills connected to course *c*.

## Results

### Course network analysis

To illustrate the linkage between VET courses through their associated work skills, we first constructed the VET course network by projecting the bipartite network of courses and skills onto a monopartite course network. Our experiments show that the network maintains structural stability across different embedding settings, such as maximum token length and threshold values, demonstrating its robustness and ability to adjust. Furthermore, we assessed the impact of padding on the network structure by calculating the cosine similarity between course vectors generated with and without padding. The average similarity across all courses converged to 1.0, with a standard deviation of $7.41\;\times \;10^{-8}$, indicating that the structural properties of the network are unaffected by padding.

The projected VET course network successfully preserves the textual attributes of the analyzed entities, maintaining relationships among skill and course entities. The clustering of courses by their categories highlights how the network retains the characteristics of entities (see Fig 1). The finding can be further backed up by the average path length between the top 10 VET sectors, which reflects the proximity between related sectors (see S3 Fig). For example, sectors associated with human management, such as education and HR, are closely interconnected. Likewise, the business management sector exhibits relative proximity to other relevant sectors such as sales, marketing, and information technology.

The degree centrality analysis revealed that certain courses act as major hubs within the network, with a maximum degree of 538 and an average degree of 309.72 among the top 10% of nodes [52]. When examining the top 10% of nodes by degree, the most frequent course label was “Workplace Safety and Health" (28.94%). Other prominent labels include leadership (9.65%), HR (8.16%), information (6.86%), business (5.94%), education (5.57%), and food (4.27%). These findings highlight the role these key areas play in connecting various vocational skills, providing valuable insights into the dominant skill domains within the VET landscape.

**Fig 2:**
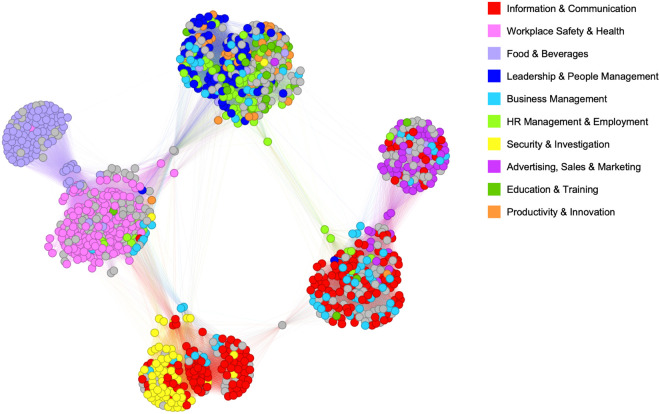
The k–core subgraph with *k* = 100. The color of the nodes indicates different VET course sectors.

The *k*-core decomposition, removing nodes with fewer connections than the specified *k* [53], supports the presence of distinct clusters represented by the course categories. In Fig 2, six separate clusters are found for *k* = 100, representing (1) sales, (2) mix of information and business, (3) mix of information and security, (4) safety & health, (5) food, and (6) mix of HR and leadership.

### Measuring course influence in Singapore VET market

We propose a metric called “course influence” to quantify the potential implication of individual VET courses. The metric aims to assess the potential influence of a VET course on work skills, given the number of similar courses that possibly provide similar educational content. On the one hand, the coverage *C*_*c*_ of a course *c* counts the number of connected skills in the course-skill network, which represents the abilities for employees to acquire for school-job transition or career development, thereby reflecting labor market demand. This is analogous to how the prior study infers skill demand based on co-occurrence patterns in job requirements, but adapted to the VET context [54]. On the other hand, the degree centrality (*k*_*c*_) of a course, counting the number of other courses with similar descriptions, denotes the complementarity between VET courses, indicating the overlapping supply within the educational market. This is conceptually parallel to how previous research constructs skill networks to infer labor market dynamics from the supply side [21]. Just as co-occurring skills within individual workers reveal common patterns of skill supply in the labor market, the extent of overlapping skills covered by courses reflects the saturation of VET supply.

Then, the course influence *CI*_*c*_ is calculated by dividing each course’s coverage by its degree centrality as

$$CI_{c}=\frac{C_{c}}{k_{c}}.$$ (6)

The approach accounts for the redundancy among similar VET courses while highlighting the number of distinct skills each course covers. By balancing the supply of overlapping SkillsFuture courses with the breadth of skills provided, the metric ensures a more precise representation of the relationship between labor market demand and educational offerings. The index exhibits an exponential distribution for our dataset (see S4 Fig).

### Course influence and future economy

The suggested course influence index of VET courses needs to be explained with respect to their diversity in targeted skills since determining the types and number of skills is an essential step for designing curricula and course content. We incorporated VET skill diversity and VET transferability in our regression model to examine their relationship with the course influence of VET courses. We constructed Ordinary Least Squares (OLS) regression models with course influence as the dependent variable to delve deeper into this question. Model (1) integrates variables for the diversity and transferability associated with four distinct future economies as independent variables. Model (2) utilizes only those variables identified as significant from Model (1). Model (3) substitutes the diversity factor with salary to examine the relationship between salary and course influence. The median salary for each course was calculated as the median monthly income from related occupations within the course-skill network. Lastly, Model (4) includes the same variables as the previous models but excludes the information of VET sectors to compare the results with and without this control. As the course influence index follows an exponential distribution, a log transformation was applied before model fitting. The median salary was also log-transformed to stabilize its distribution.

Table 1 presents the results of regression models examining key factors associated with course influence. The findings reveal two main factors: transferability for the Fourth Industrial Revolution and low skill diversity. From Model (1), only the transferability value related to the Fourth Industrial Revolution economy is positively associated with course influence (1.2592^***^) among the four transferability indices. This indicates that VET courses related to skills creating job opportunities for the Fourth Industrial Revolution economy tend to have greater potential implications by covering more skills effectively. In contrast, the transferability for the digital, care, and green economies was not found to be statistically significant, suggesting that courses related to these economies do not exhibit a significant difference in the number of work skills covered. Additionally, the negative coefficient for skill diversity (–3.6741^***^) indicates that courses spanning different skill sectors, i.e., covering more general skills, are likely to cover fewer skills.

**Table 1:** Regression analysis on VET course influence. VET course labels are under control within the model.

	*Dependent variable: log(Course Influence)*			
	(1)	(2)	(3)	(4)
Intercept	1.4265^***^	1.4107^***^	–3.1383^***^	1.1741^***^
	(0.217)	(0.217)	(0.399)	(0.212)
Transferability (4th IR)	1.2592^***^	1.2483^***^	1.8807^***^	1.3755^***^
	(0.153)	(0.153)	(0.169)	(0.154)
Transferability (Digital)	–0.1338		–0.019	–0.1266
	(0.131)		(0.146)	(0.128)
Transferability (Care)	–0.0573		0.0522	–0.3458^***^
	(0.134)		(0.152)	(0.130)
Transferability (Green)	0.1836		0.1358	0.3644^*^
	(0.207)		(0.241)	(0.209)
Skill Diversity	–3.6741^***^	–3.6627^***^		–3.887^***^
	(0.223)	(0.223)		(0.222)
log(Median Salary)			0.1262^***^	
			(0.043)	
VET sector FE (fixed effect)	Yes	Yes	Yes	No
Observations	3571	3571	4999	3571
${\text{R}}^{2}$	0.220	0.219	0.158	0.115
Adjusted ${\text{R}}^{2}$	0.207	0.207	0.148	0.114

^***^p<0.01; ^**^p<0.05; ^*^p<0.1; standard errors are in parentheses.

Model (2), which includes only the highly significant variables from the previous model, confirms the robustness of Model (1). Model (3) investigates the relationship between the log-transformed median salary and course influence. The coefficient for log median salary is 0.1262^***^, indicating a very statistically significant positive relationship. This result suggests that courses with higher influence scores are associated with higher median salaries. Finally, Model (4) repeats the analysis without controlling for VET sectors, showing the robustness of the results for this control.

Variance inflation factors were examined to assess the potential multicollinearity among the independent variables. Excluding the constant, the VIF values for transferability for four economies and skill diversity were all close to 1, indicating that multicollinearity is not a concern in our models (see S2 Table).

### Explaining course influence with VET specificity

**Fig 3:**
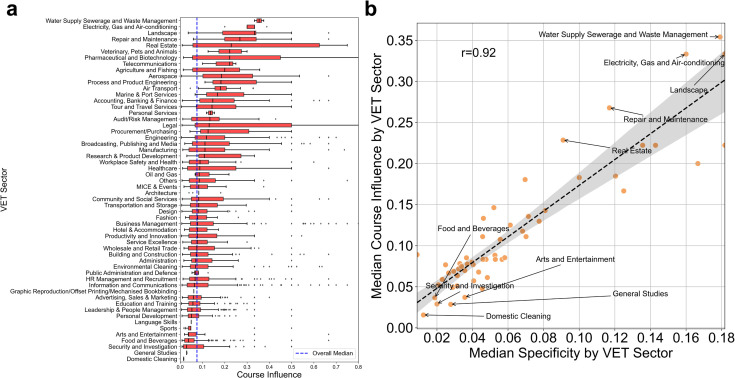
Course influence and VET specificity. **a**. Comparison of course influence across different VET sectors. The box plot includes the median, quartiles, and 1.5 interquartile range (IQR) of course influence values for each VET sector. The dashed line indicates the overall median value of course influence over all sectors. **b**. The relationship between the specificity and course influence at the VET sector level. The horizontal axis represents the median specificity for each VET sector, while the vertical axis represents its median course influence. The text labels and the shade denote the name of VET sectors and the confidence interval of the simple linear regression, respectively.

So far, we have focused on the relationship between economic variables and the influence of individual courses. Analyzing the course influence at the level of VET sectors can provide insights into the supply and demand of courses for each sector. Fig 3 reveals significant differences in the median values of the course influence across various VET sectors. The sectors with relatively high course influence include “Waste Management", “Landscape", “Electricity and Air-conditioning ", and “Repair and Maintenance", while “Domestic Cleaning", “Security and Investigation", “Arts and Entertainment", “General Studies" and “Food and Beverages" exhibit low course influence.

The variation in course influence across VET sectors is seemingly associated with each sector’s specificity, i.e., the extent to which a VET course specializes in specific working skill groups. To examine this, we define VET specificity by adopting the ubiquity of related skills (see Materials and Methods for details), and compare the median specificity with the median course influence across different VET sectors. Fig 3b shows a strong correlation between the specificity and course influence (*r*=0.92). This finding aligns with the result from the regression analysis, where lower diversity is associated with higher course influence. Both findings imply that when developing new VET courses, specializing in particular target skills as learning objectives can be an effective strategy to maximize the influence.

### Course supply by occupation in the Singapore labor market

The observed imbalance in course influence across VET sectors implies potential oversupply and undersupply of educational opportunities for different occupations in Singapore. To examine the distribution of VET course supply across occupation sectors, we employ an “occupational course supply” (namely, OCS) index which captures the number of courses offered for a particular occupation relative to the average over the entire occupations as:

$$OCS_{o}=\frac{C_{o}}{\overset{―}{C}},$$ (7)

where:

*C*_*o*_ = number of courses offered for occupation *o*,

$\overset{―}{C}$ = average of the number of courses offered for occupations.

**Fig 4:**
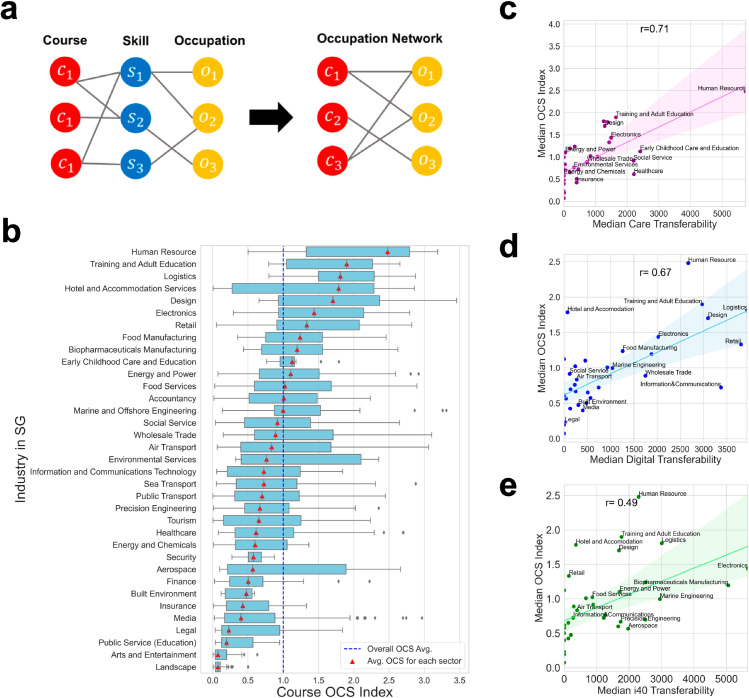
Occupational course supply and comparison with transferability. **a**. Construction of the course-occupation network from the tripartite network of courses, skills, and occupations. Links are connected for entities with embedding similarity higher than 0.6. **b**. OCS index for each occupation sector in Singapore. The average for each sector is denoted by a red triangle, and the blue dashed line indicates the average of the OCS index over all occupations. The box plot denotes the median, quartiles, and 1.5 IQR. **c-e**. Comparison of the average OCS index and average transferability for care economies (c), digital economies (d), and industrial 4.0 economies (e) across occupation sectors. The colored line and shade represent the simple regression plot and its confidence interval, respectively.

We obtained the number of courses offered for each occupation, *C*_*o*_, from the bipartite network of VET courses and occupations as we computed the number of skills covered by a course (i.e., *C*_*c*_) from the network of courses and skills. To get this bipartite network of courses and occupations, we constructed a tripartite network of courses, skills, and occupations by adding the skill-course bipartite network to the course-skill network (see Fig 4a for construction of the course-occupation network). We linked skills and occupations in the same way as the course-skill network, utilizing their embedding vectors’ textual similarity over 0.6. The course-occupation network was built from the tripartite network by connecting a link between a course and an occupation that can be reached through a skill. Then, we get *C*_*o*_ by counting each occupation’s degree, i.e., the number of courses linked to the occupation, on the bipartite network.

The observed OCS index shows that occupational sectors such as HR, Training and Education, logistics, and design tend to have more courses, while other sectors like Built Environment, Legal, Public Service, Arts and Entertainment, and Landscape have relatively fewer courses. This suggests that occupation sectors with lower OCS indices benefit less from the SkillsFuture program with fewer courses and may require more VET course supply.

The imbalance captured by the suggested index highlights the disparities in the provision of VET. Sectors with fewer courses may struggle with the development of the workforce and skill acquisition, making it more challenging to adapt to evolving economic demands. On the other hand, sectors with a higher concentration of courses may benefit from a stronger pipeline of skilled workers, reinforcing existing labor market structures.

The OCS index and occupational transferability at the sector level show the highest correlation for care economies (*r* = 0.71), followed by the digital (*r* = 0.67) and the fourth industrial revolution (*r* = 0.49) economies (see Fig 4c, 4d, and 4e). The green transferability is not significantly correlated with the OCS index, despite the growing importance of sustainable development, demonstrating that the VET market has not overly saturated this sector with training courses. As such, the SkillsFuture VET market exhibits a disproportionate concentration in particular jobs.

## Discussion

To assess the influence of Vocational Education and Training in Singapore’s labor market, this study utilized a course/skill/occupation network based on textual similarity. Leveraging the constructed network, we quantified each VET course’s influence as the ratio of the number of skills it covers to its degree centrality in the monopartite network, allowing for a balanced assessment of its impact. This approach allowed us to find that courses covering specific skills tended to exhibit high influence, as measured by the suggested specificity index. We also examined the imbalance of educational opportunities across occupations by constructing a course-occupation network, which links VET courses to occupations based on shared skills. The network was derived from a tripartite structure connecting courses, skills, and occupations. Skill-occupation relationships were established using cosine similarity with a threshold of 0.6, identical to the course-skill network. Our analysis highlighted an imbalance in educational opportunities, characterized by a high course supply for occupations requiring general skills and those in care economies.

The use of text data in this study is particularly noteworthy due to its high accessibility as a method for quantification. This approach makes it possible to quantify some features that had not been previously quantified, despite the availability of relevant text information. We selected the all-MiniLM-L6-v2 model for document embedding, ensuring consistent input processing by applying padding and truncation, with input text longer than 256 tokens being truncated by default. The embedding technique enabled the construction of networks connecting courses, skills, and occupations by calculating the cosine similarity between embedding vectors. We expect this approach to help address data challenges in evaluating the effectiveness of VET programs. Analyzing previously untapped text data could open new avenues for education research, providing a deeper understanding of VET’s impact on skills development and labor market outcomes. Also, this method is not limited to VET; it can be applied to a wide range of education studies, offering insights into how different forms of education can influence workforce readiness and skill acquisition.

The indices proposed in this study could offer valuable contributions to existing research in several aspects. Firstly, to the authors’ best knowledge, this study appears to be among the first to holistically and quantitatively explore the complex relationships between VET programs, skills, and occupations. By doing so, it seeks to extend the scope of labor market research beyond the traditional focus on the relationship between specific skills and employment. Moreover, the study introduces a new way of measuring the effectiveness of vocational education, which has predominantly relied on qualitative research based on surveys and course reviews. Lastly, the study contributes to the context of VET evaluation by proposing the VET specificity index adopted from the ubiquity index which has been originally used in the field of economic complexity.

To ensure the robustness of our methodology, we conducted a series of sensitivity analyses. First, we compared networks built using sequence lengths of 200 and 256 tokens; the resulting degree centralities exhibited a Pearson correlation coefficient of 0.98, demonstrating that the network structure of the course network is highly robust to variations in token length. Second, we validated our padding strategy by computing cosine similarities between embeddings of the same course generated with and without padding. The average similarity converged to 1.0, confirming that padding does not substantially affect the semantic content of the text. Finally, we examined the impact of varying the similarity threshold. Although network structure metrics such as link density and giant component ratio exhibited notable variations across different threshold values, the course influence ranking remained relatively stable (see S5 Fig). These robustness tests confirm the validity of our selected parameters and demonstrate that our findings are not overly sensitive to any specific configuration.

Given the evolving landscape of future regional economies and work skills influenced by climate change, digital transformation, and pandemics, there is a growing demand for vocational education to adapt to such changes [18, 55]. For example, during the COVID-19 pandemic, a significant increase in remote or hybrid work arrangements led to a fundamental shift in working styles, necessitating new skills and tools for existing workers [56]. As a result, new vocational education and training (VET) courses have emerged to address these changing demands.

Focusing on the SkillsFuture initiative, the program has played a crucial role in Singapore’s labor market by enhancing workforce competitiveness and supporting sustainable economic growth. According to the Ministry of Education (MOE) Singapore, nearly 7,000 job placements were achieved through Workforce Singapore (WSG) and SkillsFuture Singapore (SSG) Place-and-Train programs in 2019. Additionally, over 80% of trainees also reported that they improved their job performance after completing training [57]. In this light, the methods and indices proposed by this study help assess the macroeconomic and social impacts of VET, including SkillsFuture, aiding policymakers with informed decisions.

The study is not without limitations. One limitation is its sole focus on the four future economies, without considering the entirety of occupations or industries due to the lack of data. Incorporating a more comprehensive employment dataset would enable a more refined measurement of VET’s influence in Singapore and provide a more holistic understanding of its effectiveness and contribution to the workforce and economy, considering its heterogeneity across VET sectors. Another constraint is the potential bias in the data stemming from Singapore’s unique industrial landscape and employment-population structure as a city-state, which makes it challenging to generalize our findings to other countries or regions. In addition, the analysis is limited to courses with syllabi available in English, as Chinese-language syllabi—comprising over 50% of all available courses—were excluded to reduce language-related variation in the embedding process. The exclusion may affect the generalizability of the findings, particularly in interpreting the indices defined in this study within the context of Singapore’s SkillsFuture program. When interpreting Singapore’s labor market based on the findings of this study, careful consideration should be given to the differences between courses with English syllabi and those with Chinese syllabi.

One potential direction for future research is to compare the inequality of VET across different regional levels (e.g., urban vs. rural) or at the country level (e.g., developed countries vs. developing countries). This may provide valuable insights into the effectiveness and accessibility of VET across different socio-economic contexts [58]. Although enrollment data for individual courses and corresponding career outcomes would significantly enhance our analysis by directly linking SkillsFuture courses to employment trajectories, such data is currently unavailable. Moreover, our analysis treats each VET course as an independent opportunity for skill enhancement without adjusting for variations in course duration, prerequisites, or difficulty. Future studies that incorporate detailed enrollment and outcome data, along with these course-specific adjustments, would provide a deeper understanding of the initiative’s long-term career impact and further elucidate how these courses shape workforce development and economic mobility in Singapore.

## Supporting information

S1 Fig Distribution of top 30 SkillsFuture course categories. The plot displays the frequency distribution of courses among the top 30 categories in the SkillsFuture dataset, demonstrating the wide variety of VET courses available.
(TIF)

S2 Fig Giant component fraction as a function of the similarity threshold to link entities. The plot demonstrates the ratio of nodes belonging to the giant component of the course network for each threshold. The course network was made by projecting the course-skill bipartite network where the links are connected for the text similarity higher than the given threshold.
(TIF)

S3 Fig Average path length between VET sectors within the course network. The heatmap demonstrates the average path length between the top 15 VET sectors. The lighter the plot, the farther the distance between the sectors.
(TIF)

S4 Fig Distribution of course influence. The figure displays a complementary cumulative distribution function (CCDF) plot of the course influence values. The plot demonstrates that the original course influence data follows an exponential distribution.
(TIF)

S5 FigRobustness analysis based on threshold variation.a. Network’s link density and giant component ratio change as the similarity threshold varying from 0.5 to 0.7 in increments of 0.02. **b.** Sensitivity of the course influence ranking to variations in network density. It compares the rank correlation at each threshold to that at the baseline threshold of 0.6. **c.** Sensitivity of the course influence ranking to the similarity threshold in comparison with the network structure. It plots the absolute relative changes in the course influence rank correlation (Δρ), link density (Δd), and giant component ratio (Δr), each normalized to its value at the baseline threshold of 0.6. By comparing the relative change in rank correlation to that in the giant component ratio, the figure confirms that, despite significant variations in network structure, the course influence ranking remains comparatively robust.(TIF)

S1 Table Summary statistics of course, skill, and occupation dataset. **a**. Total counts for each domain and the number of unique categories in the dataset, including courses, skills, and occupations. **b**. Key descriptive statistics for course syllabi, detailing the distribution of course durations and the token lengths of syllabi. **c**. Top 15 course categories based on their proportion of the overall dataset. **d**. Skills related to future economy sectors with both their counts and transferability metrics.
(PDF)

S2 Table Variance inflation factor of independent variables. The table presents the results of the variance inflation factor (VIF) analysis conducted on the five independent variables used in the regression models.
(PDF)
