# Supplementary figures and images for: Quantifying the influence of vocational education and training with text embedding and similarity-based networks
Source: PLoS One. 2025 Aug 21;20(8):e0329405. doi: 10.1371/journal.pone.0329405 (PMC12370024; doi:10.1371/journal.pone.0329405)

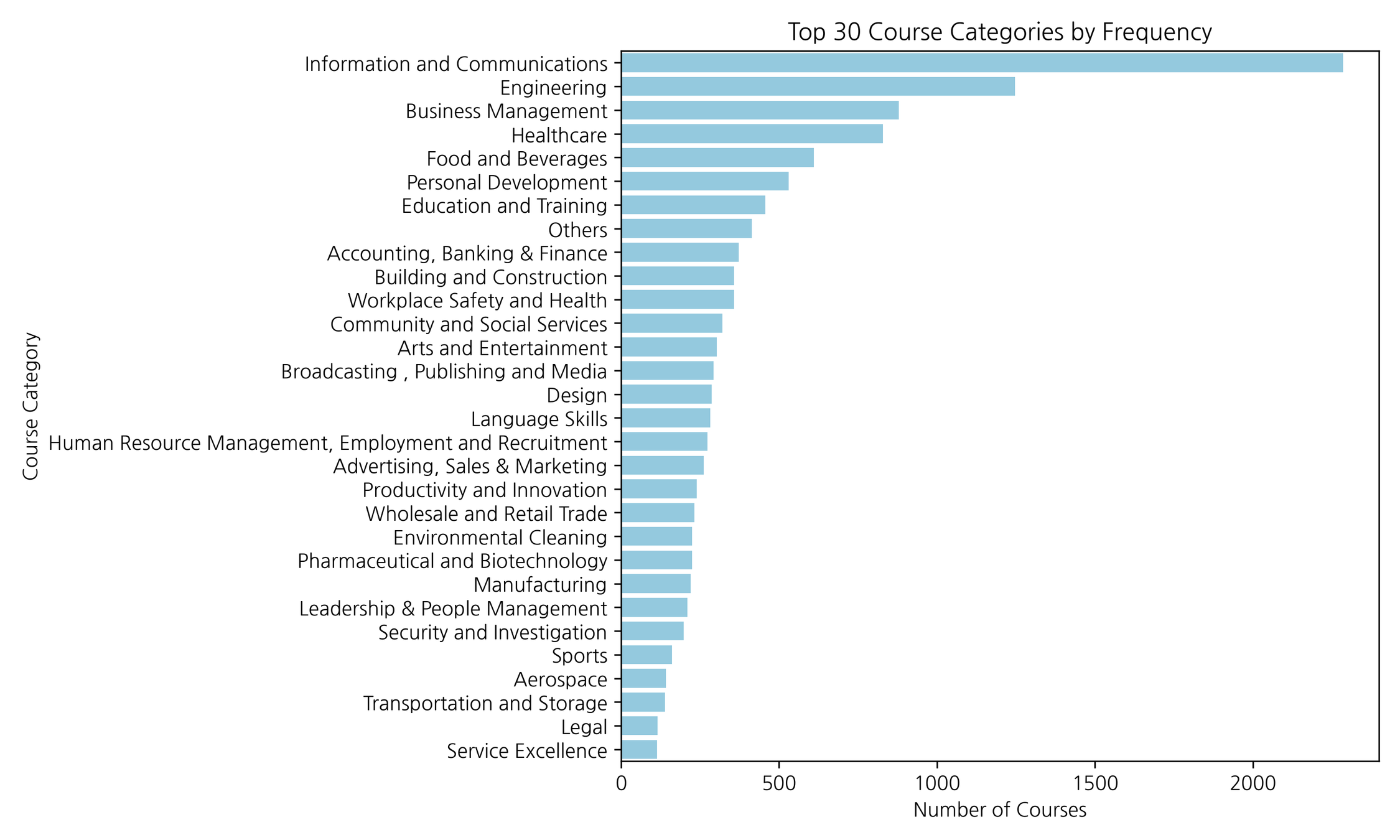

Supplement: S1 Fig — The plot displays the frequency distribution of courses among the top 30 categories in the SkillsFuture dataset, demonstrating the wide variety of VET courses available. (TIF) [file pone.0329405.s001.tif]

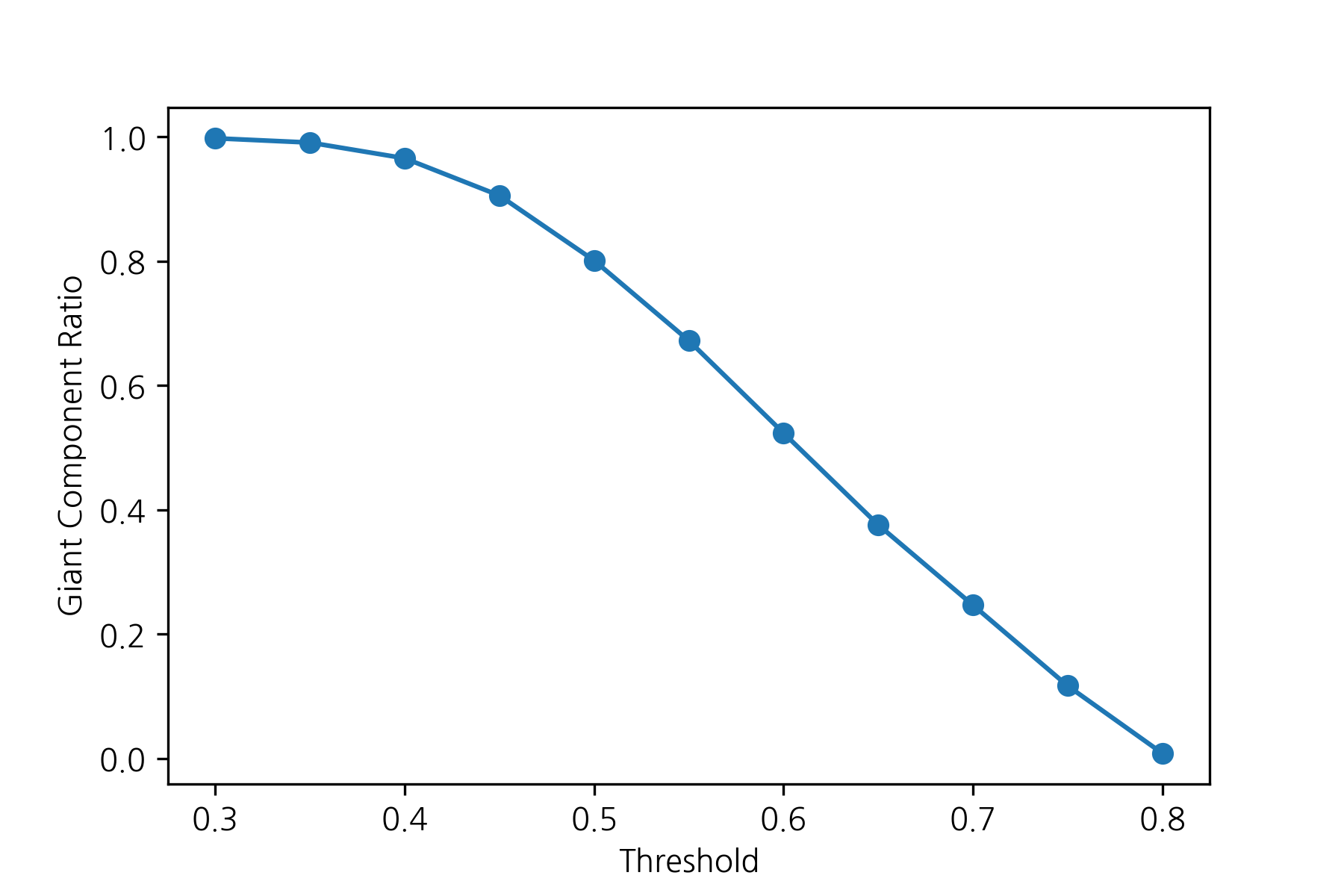

Supplement: S2 Fig — The plot demonstrates the ratio of nodes belonging to the giant component of the course network for each threshold. The course network was made by projecting the course-skill bipartite network where the links are connected for the text similarity higher than the given threshold. (TIF) [file pone.0329405.s002.tif]

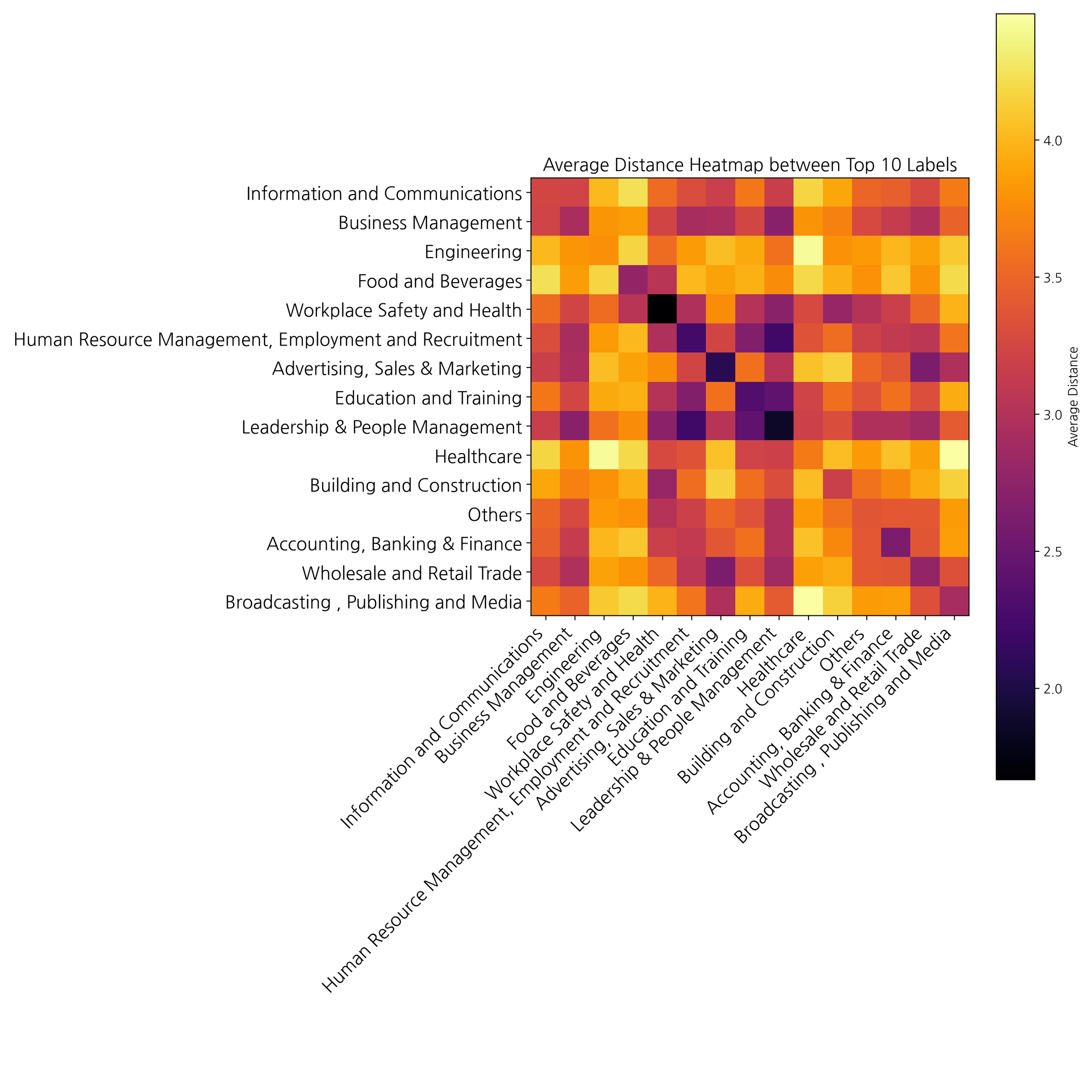

Supplement: S3 Fig — The heatmap demonstrates the average path length between the top 15 VET sectors. The lighter the plot, the farther the distance between the sectors. (TIF) [file pone.0329405.s003.tif]

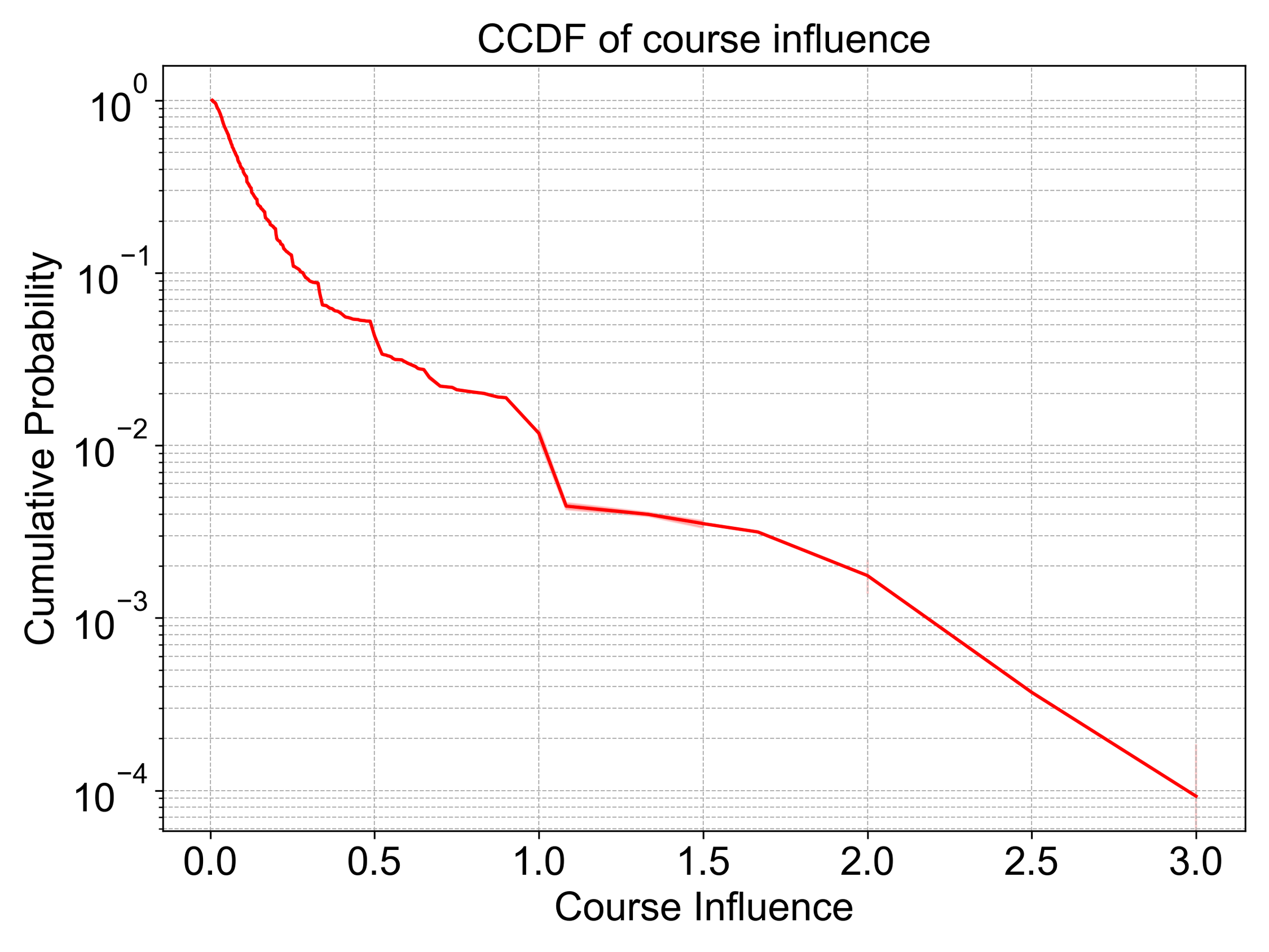

Supplement: S4 Fig — The figure displays a complementary cumulative distribution function (CCDF) plot of the course influence values. The plot demonstrates that the original course influence data follows an exponential distribution. (TIF) [file pone.0329405.s004.tif]

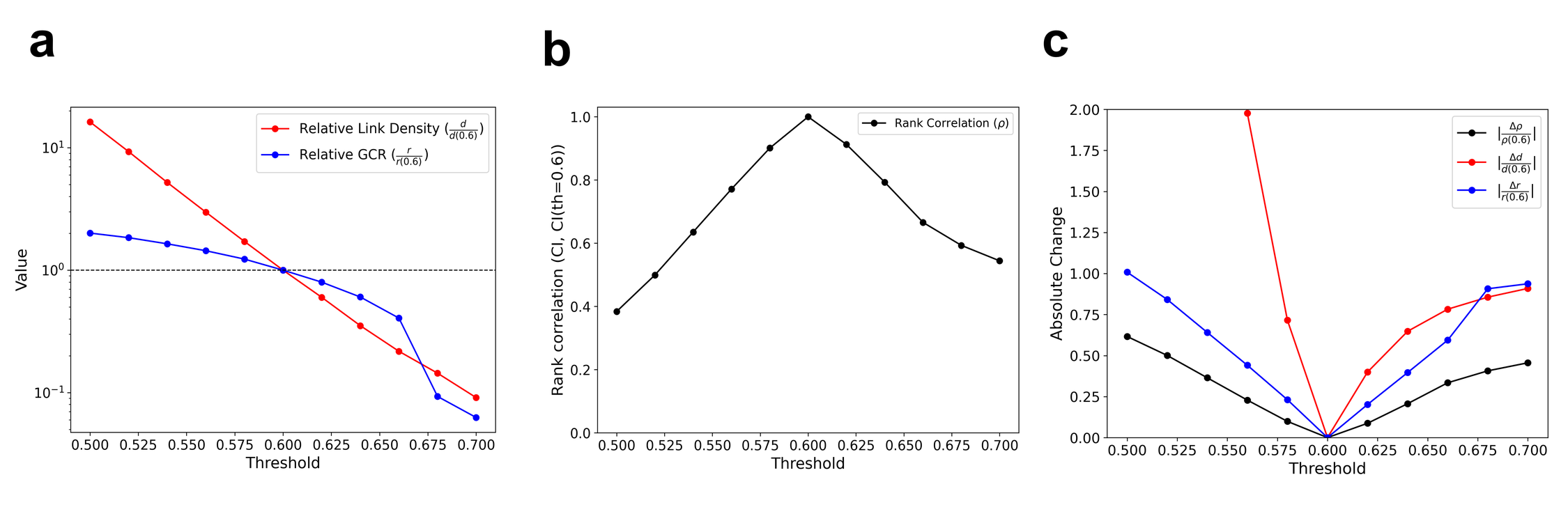

Supplement: S5 Fig — a. Network’s link density and giant component ratio change as the similarity threshold varying from 0.5 to 0.7 in increments of 0.02. b. Sensitivity of the course influence ranking to variations in network density. It compares the rank correlation at each threshold to that at the baseline threshold of 0.6. c. Sensitivity of the course influence ranking to the similarity threshold in comparison with the network structure. It plots the absolute relative changes in the course influence rank correlation (Δρ), link density (Δd), and giant component ratio (Δr), each normalized to its value at the baseline threshold of 0.6. By comparing the relative change in rank correlation to that in the giant component ratio, the figure confirms that, despite significant variations in network structure, the course influence ranking remains comparatively robust. (TIF) [file pone.0329405.s005.tif]
