# Supplementary material for: Quantifying the influence of vocational education and training with text embedding and similarity-based networks
Source: PLoS One. 2025 Aug 21;20(8):e0329405. doi: 10.1371/journal.pone.0329405 (PMC12370024; doi:10.1371/journal.pone.0329405)
Supplement: S1 Table — a. Total counts for each domain and the number of unique categories in the dataset, including courses, skills, and occupations. b. Key descriptive statistics for course syllabi, detailing the distribution of course durations and the token lengths of syllabi. c. Top 15 course categories based on their proportion of the overall dataset. d. Skills related to future economy sectors with both their counts and transferability metrics. (PDF) [file pone.0329405.s006.pdf]

S1 Table: Summary statistics of course, skill, and occupation dataset

(a) Overview of course, skill, and occupation counts

Metric	Courses	Skills	Occupations
count	13,694	2,580	1,456
category count	58	321	244

(b) Course duration and course token length distribution

Metric	Course duration	Token length per course syllabi
count	12,650	13,695
nan count	1,044	0
min	0.00	5.00
Q1	13.00	76.00
median	21.00	110.00
Q3	60.00	160.00
max	9,662.00	690.00

(c) Statistics on skill transferability for future economies

Metric	Digital	Green	Care	I4.0
Number of skills	213	46	96	143
min	1.00	1.00	1.00	1.00
Q1	35.00	12.25	22.75	17.50
median	119.00	27.00	92.50	68.00
Q3	271.00	101.50	400.25	242.50
max	843.00	419.00	934.00	909.00

(d) Distribution of course categories

Course category	Percentage (%)
Information and Communications	16.15
Engineering	9.59
Business Management	6.32
Healthcare	6.17
Food and Beverages	4.61
Personal Development	3.94
Education and Training	3.45
Others	2.97
Building and Construction	2.70
Workplace Safety and Health	2.70
Accounting, Banking & Finance	2.63
Community and Social Services	2.43
Broadcasting, Publishing and Media	2.13
Arts and Entertainment	2.09
Human Resource Management, Employment and Recruitment	1.98
Advertising, Sales & Marketing	1.93
Design	1.86
Language Skills	1.83
Productivity and Innovation	1.82
Environmental Cleaning	1.75
Wholesale and Retail Trade	1.72
Pharmaceutical and Biotechnology	1.65
Manufacturing	1.64
Leadership & People Management	1.56
Security and Investigation	1.50
Sports	1.16
Aerospace	1.12
Transportation and Storage	0.89
Service Excellence	0.87
Legal	0.87
General Studies	0.75
Agriculture and Fishing	0.62
Hotel & Accommodation	0.61
Administration	0.51
Fashion	0.51
Personal Services	0.50
Marine & Port Services	0.44
Real Estate	0.43
MICE & Events	0.38
Landscape	0.37
Veterinary, Pets and Animals	0.32
Tour and Travel Services	0.28
Research & Product Development	0.25
Repair and Maintenance	0.24
Air Transport	0.21
Electricity, Gas and Air-conditioning	0.21
Audit/Risk Management	0.20
Architecture	0.20
Telecommunications	0.17
Process and Product Engineering	0.15
Procurement/Purchasing	0.14
Water Supply Sewerage and Waste Management	0.13
Oil and Gas	0.09

**Course category**	**Percentage (%)**
Public Administration and Defence	0.09
Recreation and Attractions	0.09
Domestic Cleaning	0.05
Graphic Reproduction/Offset Printing/Mechanised Bookbinding	0.02
Insurance	0.02
