# Supplementary material for: Quantifying the influence of vocational education and training with text embedding and similarity-based networks
Source: PLoS One. 2025 Aug 21;20(8):e0329405. doi: 10.1371/journal.pone.0329405 (PMC12370024; doi:10.1371/journal.pone.0329405)
Supplement: S2 Table — The table presents the results of the variance inflation factor (VIF) analysis conducted on the five independent variables used in the regression models. (PDF) [file pone.0329405.s007.pdf]

S2 Table: Variance Inflation Factor (VIF) of Independent Variables

Variables	*VIF*	*1/VIF*
const	239.81	0.00
green_transferability	1.01	0.99
digital_transferability	1.02	0.98
care_transferability	1.02	0.98
i40_transferability	1.04	0.96
skill_diversity	1.04	0.96
